# Gangliosides: The Double-Edge Sword of Neuro-Ectodermal Derived Tumors

**DOI:** 10.3390/biom9080311

**Published:** 2019-07-27

**Authors:** Sumeyye Cavdarli, Sophie Groux-Degroote, Philippe Delannoy

**Affiliations:** Université de Lille, CNRS, UMR8576-UGSF-Unité de Glycobiologie Structurale et Fonctionnelle, F59000 Lille, France

**Keywords:** gangliosides, cancer, signal transduction, epithelial-mesenchymal transition, therapy

## Abstract

Gangliosides, the glycosphingolipids carrying one or several sialic acid residues, are mostly localized at the plasma membrane in lipid raft domains and implicated in many cellular signaling pathways mostly by interacting with tyrosine kinase receptors. Gangliosides are divided into four series according to the number of sialic acid residues, which can be also modified by *O*-acetylation. Both ganglioside expression and sialic acid modifications can be modified in pathological conditions such as cancer, which can induce either pro-cancerous or anti-cancerous effects. In this review, we summarize the specific functions of gangliosides in neuro-ectodermal derived tumors, and their roles in reprogramming the lipidomic profile of cell membrane occurring with the induction of epithelial-mesenchymal transition.

## 1. Introduction

**Figure 1:**
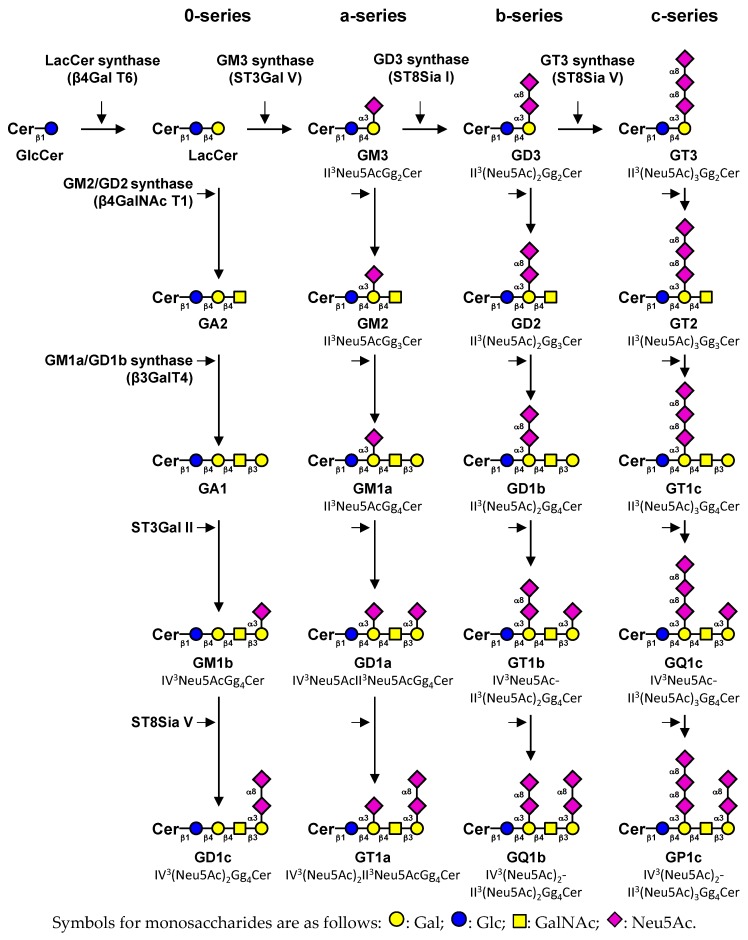
Simplified representation of ganglioside biosynthesis. Gangliosides are classified in four series according to the number of sialic acid residues linked to lactosylceramide (LacCer) [2]. The 0-series gangliosides are directly synthesized from LacCer and the precursors of other series are synthesized by specific sialyltransferases: ST3Gal V (GM3 synthase), ST8Sia I (GD3 synthase) and ST8Sia V (GT3 synthase), respectively. The elongation of precursors is performed by the sequential action of *N*-acetyl-galactosaminyltransferase (β4GalNAc T1), galactosyltransferase (β3Gal T4) and sialyltransferases (ST3Gal II and ST8Sia V). Cer, ceramide. Adapted from [6].

Gangliosides are acidic glycosphingolipids (GSL) carrying one or more sialic acid residues on their carbohydrate moieties that are mainly located in glycolipid-enriched domains, also called lipid rafts, on the outer leaflet of the plasma membrane bilayer. Raft domains are composed of cholesterol, phospholipids, and glycosphingolipids and enriched in specific proteins [1]. They engage in major cellular pathways and are involved in cell biological properties under physiological conditions. The carbohydrate part of gangliosides is constituted by glucose, galactose, *N*-acetylgalactosamine and sialic acid residues, which could exhibit numerous structural modifications such as *O*-acetylation, *N*-acetylation or sulfation. Irrespective of the elongation status of their core structure (Galβ1-3GalNAcβ1-4Galβ1-4Glcβ1-1Cer), gangliosides are classified in four series (0-, a-, b- and c-series) according to Svennerholm classification [2] in function of the number of sialic acid residues (from 0 to 3) linked to lactosylceramide. Monosialogangliosides from the a-series such as GM1, GM2 or GM3 are usually considered as simple gangliosides, whereas GD3, GD2 and GD1b (b-series gangliosides) are more complex gangliosides characterized by two sialic acid residues on their carbohydrate moieties (Figure 1). Cancer development is generally associated with glycosylation changes of glycolipids and glycoproteins expressed at the cell surface [3]. These modified carbohydrate epitopes are then defined as tumor associated carbohydrate antigens (TACA), such as GD2 or GD3 ganglioside in neuro-ectodermal derived (ND) cancers [4,5]. In this study, we review the changes in ganglioside content that are associated with tumorigenesis, their roles, and the potential therapeutic approaches that target abnormal ganglioside expression.

## 2. Expression of Gangliosides in Human Tissues

### 2.1. Monosialogangliosides Expression

Monosialogangliosides are GSL carrying only one sialic acid residue and they mainly constitute a-series gangliosides in the Svennerholm classification [2], essentially composed by GM3, GM2, and GM1. Their expression has been extensively studied during development and in different diseases, such as neurodegenerative diseases and cancers. Although there are major differences in gangliosides expression and composition between human tissues, it is widely agreed that non-neural healthy tissues mostly express monosialogangliosides, mainly GM1 [7,8]. In the central nervous system (CNS), which contains as much as 20 to 500 times more gangliosides than other tissues [9], monosialogangliosides are expressed together with more sialylated gangliosides and there is an increase in the content of gangliosides and degree of sialylation during brain development. For example, GM1, GD1a, GT1b and GQ1b were described as the major gangliosides in rat CNS [10], whereas GM3 and GD3 are the major ganglioside species described in healthy mammalian cerebral cortex [7,11].

Although the expression of monosialogangliosides is a characteristic feature of healthy tissues, they are also expressed and not necessarily down-regulated in cancer tissues and cells. Indeed, Dewald et al. showed high GM3, GM2, GM1a/b, and GD1a/b expression in MCF-7 and Hs 578T breast cancer cell lines [12]. Similarly, GM3 and GM1, which are expressed in normal CNS, are also expressed in astrocytoma and glioma cells [13,14,15]. Interestingly, GM3, which is not expressed in normal melanocytes, is detected in 60% of primary melanoma and 75% in metastatic melanoma [16]. These changes in monosialoganglioside composition seem to be highly dependent on the tumor-type and could reflect essential roles in the biology of a given cell type, contrary to disialogangliosides expression.

### 2.2. Disialogangliosides Expression

Disialogangliosides (alternatively named complex gangliosides) carry two sialic acid residues linked to lactosylceramide and constitute b-series gangliosides. Highly expressed during developmental stages, complex gangliosides are not or slightly expressed in non-neural healthy adult tissues. GD3 and GD2 are essentially expressed in CNS, peripheral nerve tissues and lymphocytes [16]. By immunohistochemical staining, Hersey et al. showed GD2 expression on T cells, B lymphocytes and dendritic reticular cells, whereas GD3 was mainly expressed on T cells [16]. In parallel, GD2 and GD3 are considered as TACA. The tumorigenesis process leads to the over-expression of GD3 and GD2 on neuroectoderm-derived cancers and sarcoma where they are considered as oncofetal markers. GD3 and GD2 are over-expressed in osteosarcoma (OS) cell lines and biopsies from patients [17,18], in leiomyosarcoma [19], in melanoma [20], in small cell lung cancer (SCLC) [21], in glioma [22] and in breast cancer (BC) [23]. GD2 is also expressed ats various rates depending on the cancer type: 25% in rhabdomyosarcoma [24,25], 59% in BC [26] and 96% in neuroblastoma (NB) tumors [27].

*O*-acetylated gangliosides are expressed in healthy tissues during developmental stages and reappear with tumorigenesis. Interestingly, *O*-acetylated GD3 (OAcGD3) is expressed in acute lymphoblastic leukemia [28] and in regenerating peripheral nerve fibers in adult rats [29]. Furthermore, OAcGD3 is expressed in benign proliferative breast lesions, and its expression increased in invasive ductal and lobular breast carcinomas [23,30], in acute lymphoblastic leukemia [31,32], in SCLC [33] and in glioblastoma (GB) [34]. *O*-acetylated GD2 (OAcGD2) expression is extensively studied in ND tumors. Alvarez-Rueda and coworkers detected OAcGD2 expression in 100% of NB, 75% of SCLC, 50% of melanoma, and 33% of renal carcinoma tissues [35]. OAcGD2 expression was established in NB, glioma, SCLC, and BC cell lines [36,37,38,39] whether OAcGD2 was absent in peripheral nerve fibers and healthy tissues [35,39].

## 3. Gangliosides Involved in Cell Fate

### 3.1. Gangliosides as Essential Components for The Maintenance of Cell Signaling

Specific glycosyltransferases (GT) are involved in the transfer of monosaccharides residues in a stepwise manner on ceramide moieties for the biosynthesis of gangliosides. GT are important tools to identify the function of specific gangliosides. Many strategies have been employed to decipher the role of gangliosides in physiology and pathologies, such as exogenous treatment by gangliosides, or over-expression or depletion of specific glycosyltransferases. The complete depletion of gangliosides was performed in the *b4galnt1/St3gal5* double knockout mice for GM2 and GM3 synthase. CNS degeneration occurred in these mice exhibiting axonal degeneration, vacuolated oligodendrocytes and abnormal axon-glia interactions [40].

In addition, gangliosides are enriched in lipid rafts, where they can modulate intrinsic and extrinsic cell signaling processes by *cis*- and *trans*- interactions with receptors tyrosine kinase (RTK) and/or the microenvironment [6]. These interactions, which are part of the biology of normal cells, can be modified in cancer cells, in which alterations of ganglioside pattern activate or inhibit modulate RTK-associated downstream signaling pathways. In that way, GM1 and GM3 expression are associated with a protective role against cancer [16,41], whereas GD3 and GD2 have a pro-tumoral role [6,42,43]. These data highlight the role of gangliosides as double-edge sword for the maintenance of cell homeostasis and for the positive or negative regulation of malignant properties of cancer cells.

### 3.2. The Anti-Tumoral Role of Monosialogangliosides in ND Tumors

**Figure 2:**
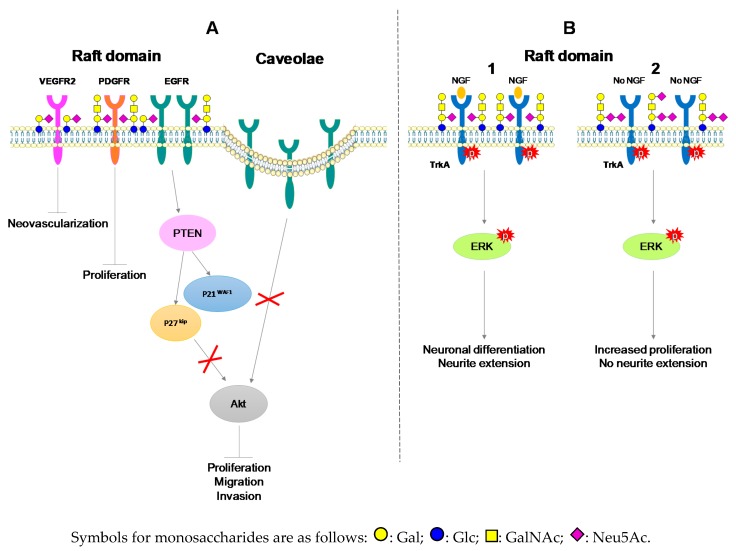
Interactions of gangliosides with growth factor receptors in neuro-ectodermal derived (ND) cancers. (**A**) Negative regulation of malignant properties of cancers cells through GM1 and GM3 interaction with growth factor receptor. (**B**) Positive regulation of malignant properties of neuronal cells through interactions of TrkA receptor with GM1 in the presence of NGF (1) or with GD1b and GT1b in the absence of NGF (2) [53].

Hanahan and Weinberg defined the hallmarks of cancer cells, which have to be negatively regulated for the inhibition of malignant properties [44], especially through the inhibition of proliferation, migration and invading capacities of cells. GM3 and GM1 induced the inhibition of cell proliferation in glioma [15], epidermoid carcinoma [45], NB [46] and astrocytoma [14]. Cell growth inhibition takes place in concert with GFR inactivation and apoptosis induction. Mirkin et al. showed that GD1a treatment had a tendency to inhibit NB cell proliferation, while GT1b treatment was more efficient on suppressing the phosphorylation of EGFR, and GM3 treatment had effects on both parameters [46]. EGFR binds to GM3 through carbohydrate-carbohydrate interactions with N-linked glycans having multiple GlcNAc termini of EGFR [47]. Besides, exogenous addition of GM1 to high-density BC cells inhibits proliferation through delocalization of EGFR from raft domains to caveolae [48,49]. GM1 interacts with platelet derived growth receptor (PDGFR), decreasing its activation in raft domains and reducing Swiss 3T3 cell line proliferation [50]. GM3 enhanced the expression of the cyclin-dependent kinase (Cdk) inhibitor p27^kip1^ [14] and CDK inhibitor p21^WAF1^ expression through phosphatase and tensin homolog deleted on chromosome 10 (PTEN), which inactivate PI3K/Akt signaling [37]. GM3 treatment also blocked dimerization of Vascular Endothelial Growth Factor Receptor-2 (VEGFR2) and its activation in vitro and neovascularization in vivo including in chick chorioallantoic membrane or matrigel plus assay [51] (Figure 2A).

### 3.3. Monosialogangliosides as Enhancers of Tumorigenesis

Monosialogangliosides do not exhibit a unique anti-tumor role in cancer development. In NB or GB, neuronal differentiation and growth are the major properties acquired during the tumorigenic process. The rat pheochromocytoma PC12 cell line is widely used as a differentiation model of neuronal cells after NGF stimulation. GM1 treatment enhanced the NGF effect on neuronal differentiation by directly interacting with TrkA and activating its autophosphorylation [52]. In that case, added GM1 did not change signal transduction or the fate of PC12 cells but just regulated the reactivity of Trk receptor to NGF.

PC12 cells transfected with GD3 synthase (PC12 GD3S+) expressed complex gangliosides GD1b and GT1b rather than GM1. In PC12 GD3S+ cells, TrkA dimerized and was constitutively activated without NGF stimulation, activating in turn the phosphorylation of ERK1/ERK2. PC12 GD3S+ cells exhibited enhanced growth but showed unresponsiveness to NGF regarding neuronal differentiation [53]. PC12 GD3S+ cells recovered neurite extension and TrkA autophosphorylation after GM1 treatment, showing the regulatory role of gangliosides in cell differentiation and proliferation [53] (Figure 2B).

The modulation of GM3 synthase expression has been performed in murine BC cell lines 4T1 or 67NR. The over-expression of GM3 synthase in 67NR induces higher GM3 and GD3 expression, which promoted migration, invasion, anchorage independent growth in vitro and lung metastasis in vivo. The silencing of GM3 synthase in 4T1 inhibited all these acquired properties without affecting cell growth [54]. In conclusion, GM1 and GM3 have been associated either with pro- or anti-cancerous properties, depending on the cell or tissue of interest. Nevertheless, it seems clear that the pro-tumorigenic effect of monosialogangliosides is essentially due to the co-expression of more complex gangliosides.

### 3.4. The Pro-Tumoral Role of Cell Membrane GD2 and GD3

On the contrary, GD3 and GD2 were shown to have mainly a pro-tumoral role in ND cancers. GD3 expression and self-renewal capacities in mice neural stem cells or in GB neurospheres have been demonstrated [55,56]. Wang et al. showed maintained EGFR activation by GD3, leading to the promotion EGF/ERK signaling and enhancing self-renewal capacity in mice neural stem cells [55]. In a similar manner, in neurospheres, high GD3 expression correlated with EGFR activation, increased stemness genes expression and self-renewal ability in glioblastoma multiforme [56]. In breast cancer, co-immunoprecipitation and proximity ligation assay showed colocalization of EGFR and GD3 [57], as well as EGFR activation by GD3 in cell membrane, avoiding the EGFR lysosomal degradation process [55]. In that way, GD3 seems to have an opposite role compared to GM3 regarding EGFR activation.

**Figure 3:**
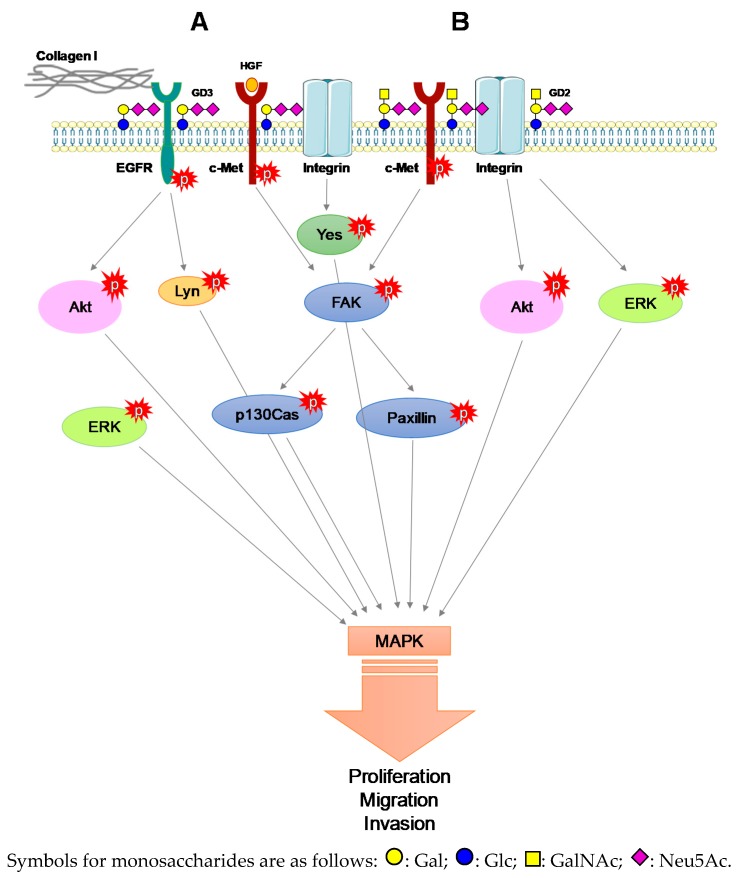
GD2- and GD3-associated MAPK signaling activation. (**A**) GD3-associated c-Met, EGFR and collagen I activation of MAPK signaling. (**B**) GD2-associated c-Met constitutive activation and integrin activation induce downstream activation of MAPK signaling.

GD3 synthase over-expression in tumor cell lines with low expression of b-series gangliosides leads to GD2 over-expression, which increases cell proliferation in SCLC [21]. GD2 was associated with integrin-β1 and c-Met receptor [57]. Carbohydrate-carbohydrate interactions between GD2 and c-Met induced its constitutive activation even in the absence of HGF [58]. In a starved medium, c-Met constitutive activation enhanced proliferation and migration of BC cell lines over-expressing GD3 synthase [59,60]. GD3 can also activate c-Met in melanoma cells. c-Met activation by GD3 depends on HGF and collagen type-I co-stimulation in GD3 synthase over-expressing SK-MEL-28 cell lines. The activation of c-Met by the tripartite GD3-HGF-collagen type I activated in turn PI3K/Akt and MEK-ERK signaling pathway, proliferation and invasion [61]. High expression of GD3 in SK-MEL-28 GD3+ cell line enhanced phosphorylation of adaptors molecules such focal adhesion kinase (FAK) and paxillin after serum treatment [42]. Integrin-β1 was essential in the maintenance of the malignant effect induced by GD3 in melanoma. Indeed, GD3 expression induced integrin clustering, enhancing phosphorylation of adaptor molecules such FAK, Paxillin and Yes, enforcing in turn invasion, motility and proliferation [62,63,64]. GD2 formed a trimeric complex with integrin-β1 and FAK and activated MAPK signaling [43]. In OS, GD2 and GD3 expression correlated with increased invasion and motility signals, with no modification in cell proliferation. GD3 synthase over-expressing OS cells exhibited strong phosphorylation of FAK and Lyn among Src family kinases enforcing paxillin activation [17], which is the key structure forming the linkage between ECM and actin skeleton. Co-immunoprecipitation experiments conducted on rat brain tissue or engineered CHO cell line over-expressing GD3 synthase demonstrated direct interactions between GD3 and Lyn, activating Lyn and the downstream MAPK signaling pathway [65]. GD3 and GD2 enforced proliferation, motility, invasion of ND tumors in vitro and in vivo (Figure 3). In that way, GD3 and GD2 confer resistance to apoptosis.

### 3.5. Alternative Roles of O-acetylated Derivatives of GD3 and GD2 in ND Tumors

Depletion of b-series gangliosides in GD3 synthase null mice showed reduced regeneration after hypoglossal nerve lesions. Complex gangliosides are essential for lesion regeneration [66,67,68]. Indeed, OAcGD3 was re-expressed for a short time period after sciatic nerve crush in adult rats. OAcGD3 expression was spatio-temporally correlated to axon regrowth through the lesion site. These results suggest that OAcGD3 plays a role in the regeneration of axon fibers after peripheral nerve lesions [29]. We can assume that b-series gangliosides are critical in the repair of damaged neural tissues in vivo [65]. After *de novo* synthesis, GD3 could relocate to the mitochondrial membrane and contribute with intracellular calcium to the opening of mitochondrial pore complex and induce the release of apoptogenic factors such as reactive oxygen species (ROS), cytochrome c, and caspase 9 [69,70]. Endogenous GD3 sustains an apoptotic role in contrast to OAcGD3 or raft domain-localized GD3. In GM2/GD2 synthase KO mice, GM3, GD3 and OAcGD3 accumulate in the brain [71]. In the same manner, HEK-293 cells over-expressing GD3 synthase exhibit higher levels of OAcGD3. Both GB primary tissues and U811 GB cells accumulate OAcGD3, which promotes the survival of GB cells, protecting them from GD3 induced mitochondrial apoptosis [34,72,73].

To date, the mechanisms of ganglioside *O*-acetylation remain obscure. However, Arming et al. identified human *CASD1* gene (Cas 1 domain containing 1) sharing sequence similarity to *Cas1p* (Capsule Synthesis 1) in *Cryptococcus neoformans* encoding for a sialate *O*-acetyltransferase (SOAT). CASD1 transfected COS7 cells exhibit high expression of OAcGD3 [74]. Baumann et al. suggest that CASD1 acts on the activated sialic acid donor, CMP-Neu5Ac, and not on the ganglioside itself [75]. Yet, the involvement of specific SOAT in ganglioside *O*-acetylation remains unclear. *O*-acetylated GD2 is barely studied among ganglioside species. Recently, Cochonneau et al. showed a proliferative role of *O*-acetylated GD2 in NB and melanoma cell lines and in SCID mice using anti-OAcGD2 monoclonal antibody [36].

### 3.6. Gangliosides Are EMT Modulators

The Epithelial Mesenchymal transition (EMT) is a natural process by which an epithelial cell is subjected to several morphological changes leading to the acquisition of mesenchymal phenotype with the ability to metastasize. The loss of epithelial polarity is therefore one of the main characteristics of EMT. In that sense, Gocht et al. have shown a link between abnormal OAcGD3 localization and the loss of epithelial polarity in BC [30]. Moreover, EMT and metastasis are regulated by several signaling molecules in the microenvironment such as TGFβ or TNF. TGFβ induces GM3 synthase expression in human epithelial lens cell HLE-B3, which exhibit higher GM3 expression. GM3 interacts with TGFβR and promotes HLE-B3 migration and EMT [76]. Furthermore, cells undergoing EMT acquired self-renewal capabilities and stem cell properties exhibiting markers such CD44^high^ CD24^low^ [77]. Battula et al. demonstrated that a small proportion of BC cells that exhibit co-expression of GD3 synthase, ganglioside GD2 and CD44^high^ CD24^low^ were capable of mammosphere formation and tumor initiation [78]. GD2 is considered as a BC stem cell marker and promotes tumorigenesis. GD2 expression is also linked to EMT induction. In EMT-induced HMLE cells, GD3 synthase and GD2 expression increase [78]. In addition, EMT is regulated by FOXC2 transcription factor, which binds directly to GD3 synthase promoter region [57]. There is therefore a feedback loop between EMT and GD3 synthase, which regulates each other’s expression and promote tumorigenesis. The intrinsic properties of cancer stem cells with enhanced self-renewal capacities lead to chemotherapy resistance.

## 4. Immune System Reactivity against Ganglioside Expression

### 4.1. Chemoresistance Supported by Gangliosides

Tumors display changes of ganglioside expression on cell membranes. These modifications lead to modulation of cell signaling pathways and to the acquisition of chemoresistant properties. Doxorubicin resistant SCLC cell line expressed higher levels of GM2, and cisplatin resistant SCLC cell line exhibited a greater amount of both GM3 and GM2 compared to the parental cell line, suggesting that the alteration of ganglioside composition may be involved in the acquisition of drug resistance [79]. GM3 over-expression by transfection on murine 3LL Lewis lung carcinoma cell line also correlated with apoptosis resistance and tumor growth [80].

The immune system plays an important role in chemotherapy response. Deactivation of the immune system is targeted by cancer cells through the inhibition of immune surveillance. The blockade of anti-tumor response in tumor microenvironment results from the combinatory effects on regulatory T-cells, myeloid derived suppressor cells, immunosuppressive dendritic cells and immune inhibitory checkpoint molecules. Tumor infiltrating lymphocytes (TILs) are mononuclear immune cells that infiltrate especially solid tumor tissues. The presence of TILs in and around the tumor is associated with improved outcome to the therapy and a better patient survival. The relationship between TILs and chemotherapy is linear, with a correlation between increasing percentage of tumor infiltration and increased sensitivity and response to therapy, improving overall patients’ survival [81,82]. Genetic or transcriptomic alteration of Ras/MAPK signaling was correlated to reduced TILs count in triple negative breast cancer (TNBC) promoting immune evasion. Additionally, GD2 and GD3 expression was demonstrated in TNBC [23]. This expression contributed to the activation of Ras-MAPK signaling pathways in ND cancers. Interestingly Loi et al. demonstrated that Ras-MAPK activity could suppress the expression of MHC-I and MHC-II in TNBC to circumvent the antigen presentation pathway. Thus, ganglioside expression activates mechanisms for cancer cell immune escape, especially GD2, which is characterized as a BC stem cell marker [81,82].

Gangliosides could be shed from tumor cells into the plasma, the serum or could be secreted via exosomes, bind to the surface of normal cells and change the lipid composition of raft domains. They often integrate cells surrounding tumors microenvironment such as fibroblasts, endothelial or immune cells. Gangliosides are involved in tumor associated T-cell activation [83]. Exosome-associated GD3 leads to the functional arrest of T-cells through their TCR in ascites fluids of ovarian cancer [84]. Purified GD3 internalization by activated T-cells initiates apoptosis by induction of ROS, accumulation of p53 and Bax, and cytochrome c release and activation of caspase-9 [65]. GD3 is also known to inhibit innate natural killer T-cells (NKT) activation through binding to CD1d presented antigens in mice and human [85].

### 4.2. Gangliosides as Therapeutic Targets for Cancer

The effect of gangliosides on cancer cells is highly dependent on the cancer type and the ganglioside of interest. Indeed, exogenous treatment of cells by purified gangliosides was extensively performed, with various results. GM3 treatment of primary cultures of high-grade human GB multiforme drastically reduced the cell number in vitro and also in a murine xenograft model, as well as in tumors from multiple ND origin such ependymomas, astrocytomas, gliomas, oligodendrogliomas, and gangliogliomas [13]. Besides, exogenous treatment of glioma by OAcGD1b or its derivative O-butyryl GD1b reduced tumor cell proliferation and cell cycle progression, potentiating the antitumoral response in xenotransplants in nude mice and intracranial allotransplants in rats [86]. Increasing the levels of GD1b by exogenous treatment or using transfection on BC cell lines induced caspase-3 and -7 mediated apoptosis. Taxane is an anti-microtubule agent inducing cell apoptosis and causing also damage to the peripheral nerves called “taxane-induced neuropathy” in BC. This adverse effect of chemotherapy is dose limiting and decreases with decreased taxane doses. A recent clinical trial has shown reduced incidence of taxane-induced neuropathy in BC patient after GM1 treatment [87].

### 4.3. Anti-Ganglioside Immunotherapy

Cancer immunotherapy is a treatment with monoclonal antibody targeting tumor-associated markers, such GD2 and GD3. A phase I clinical trial of 80 mg/m^2^ murine IgG3 R24 anti-GD3 antibody demonstrated tumor regression, Antibody-Dependant-Cell-Cytotoxicity (ADCC), Complement Dependent Cytocoxicity (CDC), and T-cell activation, in patients with malignant melanoma [88]. Recently an anti-GD2 antibody Dinutuximab (Unituxin™) mAb has been approved by Food Drug Administration (FDA) and European Medicines Agency (EMA) for the treatment of high-risk NB patients [89]. A randomized clinical trial demonstrated a significant improvement of patient outcome by therapeutic combination of chimeric antibody targeting GD2 ch14.18, interleukin-2, 13-*cis* retinoic acid and granulocytes and macrophage colony stimulating factor (GM-CSF) [90].

In SCLC, the use of monoclonal anti-GD2 antibody 14G2a induces either anoïkis by dephosphorylation of FAK, or apoptosis by p38, c-Jun terminal kinase (JNK) and caspase-3 activtion [21,43,91]. In similar manner, anti-GD2 antibody induces apoptosis by alteration of mitochondrial membrane potential, cell membrane permeability, and cell volume decrease in NB [92]. Therapeutic combination of anti-GD2 14G2a antibody with several inhibitors showed enhanced cytotoxicity efficiency. In OS, this antibody combined to endothelin A receptor antagonist (ETAR) had a greater inhibition efficiency of cell growth, invasiveness and MMP-2 activation than individual treatment [93]. In the same manner, the combination of 14G2a with either PI3K/Akt/mTOR inhibitor or aurora kinase A enhanced the cytotoxicity effect by decreasing MYCN amplification and activation of pleckstrin homology like-domain family member 1 (PHLDA1) and p53 in LAN-1, CHP-132 and IMR-32 NBL cell lines [90,94]. The synergistic effect of 14G2a anti-GD2 antibody and cisplatin has been demonstrated in SCLC and OS. This therapeutic combination strongly activated Jun Kinase (JNK), inducing cytotoxicity and apoptosis in SCLC [95] whereas it leads to the endoplasmic reticulum stress associated to apoptosis in MG-63 and Saos-2 OS cells [96].

Nevertheless, the use of anti-GD2 antibody in therapy causes several side toxicity effects such as allodynia in treated patients because of GD2 expression on healthy peripheral nerve fibers. The absence of OAcGD2 expression in healthy tissues suggests that it could be a more specific target for immunotherapy. Indeed, Terme et al., have shown that ch8B6, a chimeric antibody targeting OAcGD2 has the same effect on neuroblastoma as ch14.18 without antibody induced side effects [39].

## 5. Conclusions

Whether monosialogangliosides should be targeted would depend strongly on the tumor type, while disialogangliosides remain good targets for immunotherapy, especially in combination with drugs. Nevertheless, ganglioside composition has a great impact on the effectiveness and efficiency of treatment, which has to be adapted to the specific molecular pattern of the tumor.
